# Recurrent IgG4-related Tubulointerstitial Nephritis Successfully Treated With Obinutuzumab: A Case Report and Literature Review

**DOI:** 10.1016/j.xkme.2025.101156

**Published:** 2025-10-23

**Authors:** Shu-Hua Zhu, Du-Qun Chen, Ming-Chao Zhang, Zhe Li, Zhen Cheng

**Affiliations:** National Clinical Research Center for Kidney Diseases, Jinling Hospital, Nanjing, China

**Keywords:** B-cell depletion therapy, IgG4-related tubulointerstitial nephritis, obinutuzumab

## Abstract

Currently, Immunoglobulin G4 (IgG4)–related disease is treated with glucocorticoids alone or in combination with other immunosuppressants. Although the initial response rate is substantial, the remission rate is suboptimal, and the recurrence rate following rituximab therapy remains high. Here, we report the first patient with IgG4-related tubulointerstitial nephritis who was treated with obinutuzumab after relapse, providing additional insights for future treatment strategies. This case demonstrates that the obinutuzumab induction and maintenance regimen in patients with IgG4-related tubulointerstitial nephritis can maintain prolonged B-cell depletion, rapidly and persistently reduce inflammation and serum IgG4 levels, and reverse functional impairment in some affected organs.

Immunoglobulin G4 (IgG4)–related disease (IgG4-RD) is a relatively uncommon immune-mediated chronic inflammatory disease that potentially affects multiple organs and systems. Renal involvement typically manifests as tubulointerstitial nephritis, with a minority of patients exhibiting glomerular lesions primarily characterized by proteinuria. In severe cases, this can lead to acute or chronic renal insufficiency.^1^ Currently, glucocorticoids (GCs), either as monotherapies or in combination with other immunosuppressants, demonstrate high efficacy, yet the remission rate remains suboptimal.^2^ Rituximab (RTX) is utilized in IgG4-RD patients who do not respond to conventional therapy, who relapse during GC tapering, or who exhibit GC resistance or intolerance.^3^ However, long-term follow-up indicates a persistently high recurrence rate with this regimen.^4^ Here, we report a case of IgG4-related tubulointerstitial nephritis (IgG4-TIN) in which obinutuzumab was administered following relapse, offering additional insights for future treatment strategies in IgG4-RD.

### Case Report

A 51-year-old woman patient was admitted in September 2023 with a history of intermittent rash and elevated serum creatinine (Scr) and globulin levels for 3 years. Three years prior, she developed a dark red rash on both lower limbs without pruritus, abdominal pain, bloody stool, or joint pain. The rash resolved spontaneously within 3-4 days but recurred frequently. Laboratory tests revealed a serum immunoglobulin G level of 92.9 g/L, an IgG4 level of 38,400 mg/L, positive urinary proteinuria, and a Scr level of 202 μmol/L, leading to a diagnosis of IgG4-RD. She was treated with methylprednisolone at 24 mg/d, which led to the resolution of the rash and a decrease in Scr to 96 μmol/L, although her IgG4 level remained elevated. Methylprednisolone was gradually tapered over 2 years. In early August 2023, her Scr was 187.5 μmol/L, and her urine protein was 0.31 g/24 h. Two weeks later, she experienced large red rashes on her limbs with pruritus, irritant cough, and chest distress, which occurred 3-4 times daily. A bronchodilation test was positive, indicating mixed pulmonary ventilation dysfunction (Fig 1A). Computed tomography revealed bilateral pneumonia, with multiple enlarged lymph nodes in the bilateral hilar regions, supraclavicular fossae, axillary regions, and mediastinum. (Fig 2A-C). By the end of August, her Scr had increased to 291 μmol/L, prompting her to seek treatment at our department. Further examinations (Table 1) revealed serum IgG4 levels of 5,500 mg/L (reference range, 36 mg/L-2,090 mg/L), and renal ultrasound revealed a left/right kidney diameter of 142/136 mm. A renal biopsy was performed (Fig 3), confirming the diagnosis of IgG4-TIN, with an IgG4-RD responder index score of 8.^5^ She was treated with obinutuzumab (2 × 1,000 mg on days 1 and 14) and oral prednisone at 30 mg/d, which was reduced by 5 mg per month to a maintenance dose of 5 mg/d. The rash subsided, the cough improved, renal function normalized, and urine protein and serum IgG4 levels returned to normal. In June 2024, CD19^+^ B cells were re-examined at 2/μL, and an additional 1,000 mg of obinutuzumab was administered. A 15-month follow-up revealed no rash, stable renal function, and continuously normal urine protein and serum IgG4 levels. Cough episodes occurred 1-2 times per week, and intermittent chest tightness was observed. The bronchodilation test was positive, and obstructive pulmonary ventilation dysfunction was observed (Fig 1B). Computed tomography scans revealed a significant reduction in the number of hilar and mediastinal lymph nodes, with absorption of the lymph nodes at other sites (Fig 2a-c). The IgG4-RD responder index remained stable at 2 points.

**Figure 1:**
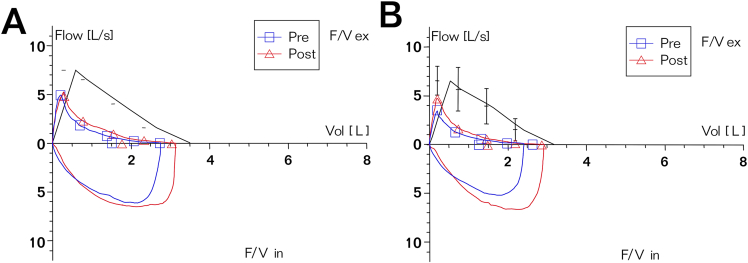
Pulmonary function curves of the patient. (A) At admission, the forced expiratory volume in 1 second (FEV1) improvement was 0.26 L, with an improvement rate of 17.51%, and the FEV1/forced vital capacity ratio was 58.25% after bronchodilator inhalation. The bronchodilation test was positive, indicating mixed ventilatory dysfunction. (B) At the 15-month follow-up, the improvement in FEV1 was 0.23 L, with an improvement rate of 18.38%, and the FEV1/forced vital capacity ratio was 52.02% after bronchodilator inhalation. The bronchodilation test remained positive, indicating obstructive ventilatory dysfunction. Abbreviation: F/V, Flow-Volume Curve.

**Figure 2:**
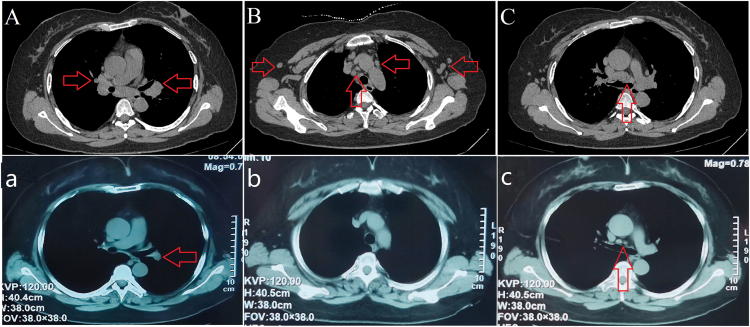
Computed tomography findings of the patient. At admission, multiple enlarged lymph nodes were observed in (A) both hilar regions, (B) supraclavicular and axillary areas, and (C) the mediastinum. After a 15-month follow-up, the (a) hilar lymph nodes and (c) mediastinal lymph nodes had significantly reduced, whereas (b) the lymph nodes at other locations had resolved.

**Table 1:** Laboratory Results of the Patient During Admission and Follow-Up for 15 Months

	On Admission	1 mo	2 mo	3 mo	6 mo	9 mo	12 mo	15 mo
WBC (×10^9^/L)	6.39	7.31	5.27	5.11	6.13	5.08	4.4	5
Eosinophils (×10^9^/L)	0.94	0.01	0.04	0.1	0.18	0.28	0.23	0.22
CRP (mg/L)	15.8	<0.5	<0.5	<0.5	1	5.1	2.7	1.7
IgG (g/L)	56.3	13.6	9.79		9.43	8.92	10.01	9.08
IgG4 (mg/L)	5500	2040	890		449	245	200	178
IgA (g/L)	2.62	2.2	2.07		1.53	1.51	1.52	1.45
IgM (g/L)	0.536	0.654	0.348		0.113	0.204	0.222	0.239
IgE (IU/mL)	205	36.7	20		3	2.7	<1.00	<1.00
RF(IU/mL)	24.5							<10
ESR(mm/h)	101							6
Complement C3 (g/L)	0.309	0.533		1.174	0.992	1.081	1.291	1.2
Complement C4 (g/L)	<0.017	0.106		0.337	0.267	0.276	0.312	0.291
ANA	1:1024							1:128
A-dsDNA	(-)							(-)
Albumin (g/L)	37.7	37.9	48.1	48.8	51.4	51	50.7	51.8
Globulin (g/L)	55.9	28.7	22.8	22.7	20.2	19.7	19.2	16.9
Scr (μmol/L)	304.1	154.7	120.2	123.8	130.8	123.7	119.34	121.11
BUN (mmol/L)	13.52	13.2	8.6	6.9	10.9	6.2	9.7	8.34
CD3^+^T (μ/L)	605	477	947	889	1162	945		988
CD4^+^T (μ/L)	246	171	338	343	424	324		356
CD8^+^T (μ/L)	322	286	573	499	669	582		605
Tregs (μ/L)	17	11	4	8	21	12		19
CD19^+^B (μ/L)	121	0	0	0	0	2		0
CD19^+^B (%)	15.88	0	0	0	0	0.12		0
eGFR (mL/min/1.73 m^2^)	15	33	45	43	40	43	45	44
Proteinuria (g/24 h)	1	0.54	0.39	0.43	0.2	0.3	0.2	0.16
Urine NAG (U/g·Cr)	32.1	18.5						6.3
Urine RBP (mg/L)	6.48	2.3		1.31	1.11	3.2	0.95	4.32
Urine NGAL (ng/mL)	182	77		30	42	77	30	

Abbreviations: ANA, antinuclear antibody; A-dsDNA, anti–double-stranded DNA antibody; BUN, blood urea nitrogen; CRP, C-reactive protein; ESR, erythrocyte sedimentation rate; eGFR, estimated glomerular filtration rate; IgG, immunoglobulin G; IgG4, immunoglobulin G4; IgA, immunoglobulin A; IgM, immunoglobulin M; IgE, immunoglobulin E; NAG, N-acetyl-b-D-glucosaminidase; NGAL, neutrophil gelatinase-associated lipocalin; RF, rheumatoid factor; RBP, retinol binding protein; Scr, serum creatinine; WBC, white blood cell.

**Figure 3:**
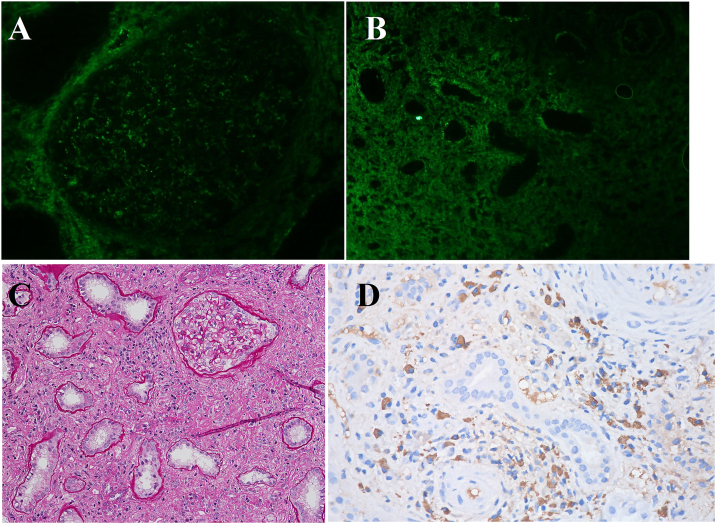
Renal pathology findings of the patient. (A) Immunofluorescence revealed that granular IgG4 deposits were diffusely distributed in the mesangial area (original magnifications, ×400). (B) Immunoglobulin G4 deposits were also observed in the renal tubular basement membrane (original magnification, ×200). (C) Light microscopy revealed global glomerular sclerosis in 50% of the glomeruli, severe chronic tubulointerstitial changes in 80%, and mild acute changes in 10%. The renal interstitium exhibited storiform fibrosis with extensive infiltration of inflammatory cells, predominantly mononuclear cells, along with a few plasma cells, neutrophils, and scattered eosinophils. Neutrophilic tubulitis was noted in several adjacent areas (periodic acid–Schiff staining; original magnification, ×200). (D) Immunohistochemistry revealed 24 IgG4-positive cells per high-power field in the renal interstitium.

## Discussion

The management of IgG4-RD prioritizes individualized treatment, aiming to reduce focal inflammation, sustain disease remission, preserve organ function, and minimize adverse effects associated with therapy. Currently, GCs are the established first-line agents for treating IgG4-RD and are applicable during both the induction of remission and the maintenance phase.^6^ GC therapy has rapid efficacy in the majority of patients, with an effectiveness rate exceeding 90%; however, the remission rate is only 65.9%, and relapses are more frequent during maintenance, with the optimal duration of GCs remaining undetermined.^7^ High baseline eosinophil counts, elevated immunoglobulin E and IgG4 levels, involvement of multiple organs, a high IgG4-RD responder index score, a history of allergies, and a low GC maintenance dose at follow-up are identified as risk factors for recurrence.^8^ Several studies have indicated that the recurrence rate among IgG4-RD patients treated with GCs in combination with other immunosuppressants is lower than that among patients receiving GCs alone. Specifically, the cumulative recurrence rates within one year for combinations of mycophenolate mofetil,^9^ leflunomide,^10^ or cyclophosphamide^11^ with GCs were 20.59%, 18.2%, and 12.0%, respectively, which were significantly lower than those reported in the GC monotherapy group (38.5%-42.4%). The overall response rates for mycophenolate mofetil,^9^ cyclophosphamide,^11^ and iguratimod^12^ combined with GCs were 76.47%, 88.8%, and 86.7%, respectively; however, the complete response rates remain suboptimal, with iguratimod achieving only 30%.

In recent years, the utilization of biologic targeted therapies in the management of IgG4-RD has garnered increasing attention. RTX, an anti-CD20 monoclonal antibody primarily employed for B-cell depletion, has demonstrated efficacy in both initial and recurrent IgG4-RD patients.^13^ Prospective studies have indicated that RTX induction therapy, without maintenance, achieves primary efficacy in 77% of patients at 12 months, with a complete response rate of 40%.^14^ However, a retrospective analysis revealed that only 30%, 55%, 39%, and 42% of patients achieved complete depletion of CD19^+^ B cells, plasma cells, naive B cells, and memory B cells, respectively, after 6 months of this regimen. The recurrence rate was as high as 70% following a median follow-up of 24 months.^15^ The administration of RTX every 6 months as maintenance therapy for remission effectively prevents relapses of IgG4-RD.^16^ For severe IgG4-RD patients with renal involvement, intensive B-cell depletion therapy can significantly increase renal function, normalize the IgG4-RD responder index, and achieve substantial improvement in clinical and histological features.^17^ Meta-analyses have demonstrated that the recurrence rates of IgG4-RD patients receiving RTX maintenance therapy are greater than those of those treated with GCs in combination with other immunosuppressants.^18^ These studies underscore the pivotal role of B cells in IgG4-RD and establish B-cell depletion therapy as a cornerstone of long-term IgG4-RD management.

Analysis of B-cell reconstitution trajectories following RTX therapy in autoimmune diseases revealed that B-cell reconstitution occurs earlier in IgG4-RD than in anti-neutrophil cytoplasmic antibody-associated vasculitis and connective tissue diseases. Elevated IgA levels and the concurrent use of GCs are associated with B-cell reconstitution.^19^ IgG4-RD may require therapies with stronger B-cell depletion capabilities.^20^ Unlike RTX, a chimeric type I anti-CD20 monoclonal antibody, obinutuzumab is a humanized type II anti-CD20 monoclonal antibody. Glycosylations in its Fc segment increase the affinity for immune effector cells, thereby augmenting antibody-dependent cellular cytotoxicity and antibody-dependent cellular phagocytosis.^21^, ^22^, ^23^ In vitro studies have shown that the antibody-dependent cellular cytotoxicity activity induced by obinutuzumab is 35-100 times greater than that induced by RTX.^22^ Preclinical research has indicated that obinutuzumab can also induce effective direct cell death and complement-dependent cytotoxicity.^23^ In chronic lymphocytic leukemia,^24^ follicular lymphoma,^25^ and membranous nephropathy,^26^ obinutuzumab has demonstrated superior efficacy compared with RTX, particularly in elderly and drug-resistant patients. Previous studies have shown that in patients with IgG4-RD–associated membranous nephropathy,^27^ retroperitoneal fibrosis, aortitis, pancreatitis,^28^ and ocular lesions,^29^ obinutuzumab rapidly reduces inflammation and serum IgG4 levels after RTX allergy, avoiding excessive use of GCs and reducing the risk of potential adverse events. However, these patients only received induction therapy, and the follow-up periods were relatively short. In some patients, serum IgG4 levels begin to increase significantly one year after obinutuzumab treatment, and long-term outcomes are lacking.

This patient exhibited a trend toward B-cell reconstitution only after 9 months of obinutuzumab induction therapy, which was significantly later than the 4-6 months reported in previous studies with RTX.^15^ Maintenance of obinutuzumab prolonged the duration of B-cell depletion and sustained remission of IgG4-RD. Importantly, a small number of residual lymph node and lung lesions suggest that acute inflammatory lesions in IgG4-RD patients can be controlled by intensive B-cell depletion therapy, but fibrotic lesions may still be challenging to reverse. To our knowledge, this patient represents the first reported case of IgG4-RD treated with a complete obinutuzumab induction and maintenance regimen in the literature, with the longest follow-up time, providing a foundation and reference for the design of subsequent clinical trials.

In summary, this case demonstrates that the obinutuzumab induction and maintenance regimen in patients with IgG4-TIN can maintain prolonged B-cell depletion, rapidly and persistently reduce inflammation and serum IgG4 levels, and reverse functional impairment in some affected organs.
